# An Automated Strategy for Unbiased Morphometric Analyses and Classifications of Growth Cones *In Vitro*

**DOI:** 10.1371/journal.pone.0140959

**Published:** 2015-10-23

**Authors:** Daryan Chitsaz, Daniel Morales, Chris Law, Artur Kania

**Affiliations:** 1 Institut de recherches cliniques de Montréal, Montréal, Canada; 2 Integrated Program in Neuroscience, McGill University, Montréal, Canada; 3 Division of Experimental Medicine, Department of Anatomy and Cell Biology and Department of Biology, McGill University, Montréal, Canada; 4 Faculté de Médecine, Université de Montréal, Montréal, Canada; Institut de la Vision, FRANCE

## Abstract

During neural circuit development, attractive or repulsive guidance cue molecules direct growth cones (GCs) to their targets by eliciting cytoskeletal remodeling, which is reflected in their morphology. The experimental power of *in vitro* neuronal cultures to assay this process and its molecular mechanisms is well established, however, a method to rapidly find and quantify multiple morphological aspects of GCs is lacking. To this end, we have developed a free, easy to use, and fully automated Fiji macro, Conographer, which accurately identifies and measures many morphological parameters of GCs in 2D explant culture images. These measurements are then subjected to principle component analysis and *k-means* clustering to mathematically classify the GCs as “collapsed” or “extended”. The morphological parameters measured for each GC are found to be significantly different between collapsed and extended GCs, and are sufficient to classify GCs as such with the same level of accuracy as human observers. Application of a known collapse-inducing ligand results in significant changes in all parameters, resulting in an increase in ‘collapsed’ GCs determined by *k-means* clustering, as expected. Our strategy provides a powerful tool for exploring the relationship between GC morphology and guidance cue signaling, which in particular will greatly facilitate high-throughput studies of the effects of drugs, gene silencing or overexpression, or any other experimental manipulation in the context of an *in vitro* axon guidance assay.

## Introduction

Since the first identification and naming of growth cones (GCs) by Cajal, studies of the morphology of these highly motile chemosensitive structures have done much to illuminate the processes governing axon guidance [1]. In particular, *in vitro* neuronal cultures have provided invaluable tools for studying how guidance cues connect neurons to their targets during development and regeneration. These powerful techniques include those which probe long-term growth direction choice as well short-term responses such as pipette assays [2–5], microcontact printed pattern assays [6], and collapse assays.

In the collapse assay, neurons are treated with a chemotropic agent, and the morphological response of GCs is frequently quantified as the percentage of GCs classified as “collapsed”, generally defined as having a smaller area and fewer filopodia and/or lamellipodia, as opposed to GCs classified as extended, which are larger and more complex [7–11]. This assay allows experimenters to take advantage of genetic or pharmacological manipulations in a highly temporally-controlled manner, to examine chemorepulsive signaling [12–14]. To assess chemotropic responses in a more quantitative fashion, it is also possible to use individual parameters such as total area rather than classifying GCs as collapsed or extended. Regardless of the strategy for quantification, the high degree of heterogeneity in GC shape generally makes it necessary to sample large numbers, requiring labor-intensive high-throughput studies. Individual metrics, meanwhile, are less biased and more amenable to automated analysis, but still depend on the observer’s definition as to what (and how much) of a structure constitutes a growth cone. While a number of algorithms can automatically or semi-automatically analyze features of neurons *in vitro*, all focus on neuronal process complexity (such as dendritic tiling or tree arborization) or axon outgrowth; to our knowledge, no freely available software currently exists which is capable of automatically finding and performing a multidimensional analysis of GCs [15–21].

Aside from the aforementioned logistical difficulties, morphology-based strategies to study GCs generally fail to attend to the complex and nuanced features of responses to chemotropic cues. Classical human-scored collapse assays generally provide only the percentage of collapsed GCs in an explant or dish, and are entirely dependent upon the experimenter’s criteria for "collapse", precluding direct comparisons of results between individuals. On the other hand, by using algorithms that measure single parameters such as GC area, the more complex features that would be taken into account by a human observer are lost. In particular, questions regarding qualitative differences in a growth cone’s short-term responses to distinct chemorepulsive cues–potentially reflecting the specific molecular pathway connecting the ligand-receptor binding event and the resulting cytoskeletal dynamics–are left unaddressed. Indeed, despite it being known that GCs are capable of taking on a number of different shapes in response to different cues and contexts, both *in vivo* and *in vitro* [11, 22–24], a proper analysis of whether subtle morphological differences in GC response can be correlated to the intracellular signaling cascades elicited by specific cues has not been performed, in part because the required automated tools have not been available

To facilitate high-throughput GC assays, we have developed Conographer, an algorithm written entirely in the ImageJ macro language that automatically detects and measures GCs in images of explanted or dissociated neurons, requiring only very simple immunohistochemical stains on the tissue. With only 3 user-determined variables, Conographer can detect GCs in images by analyzing the spatial frequencies and relative lengths of binary structures, and measure 10 different morphological parameters. While the algorithm’s strict definitions of a GC cause it to generally find fewer than humans, this constraint leads to a rate of false positives that is nearly identical to that of humans. Conographer is freely available, easy to use, and highly amenable to modifications. In this report, we describe the function of Conographer and a strategy to use its output data to mathematically assign collapse states to GCs, and demonstrate its utility in measuring changes in GC morphology brought about by Netrin-1 induced collapse.

## Materials and Methods

### Explant preparation and cultures, and collapse assay

**Fig 1:**
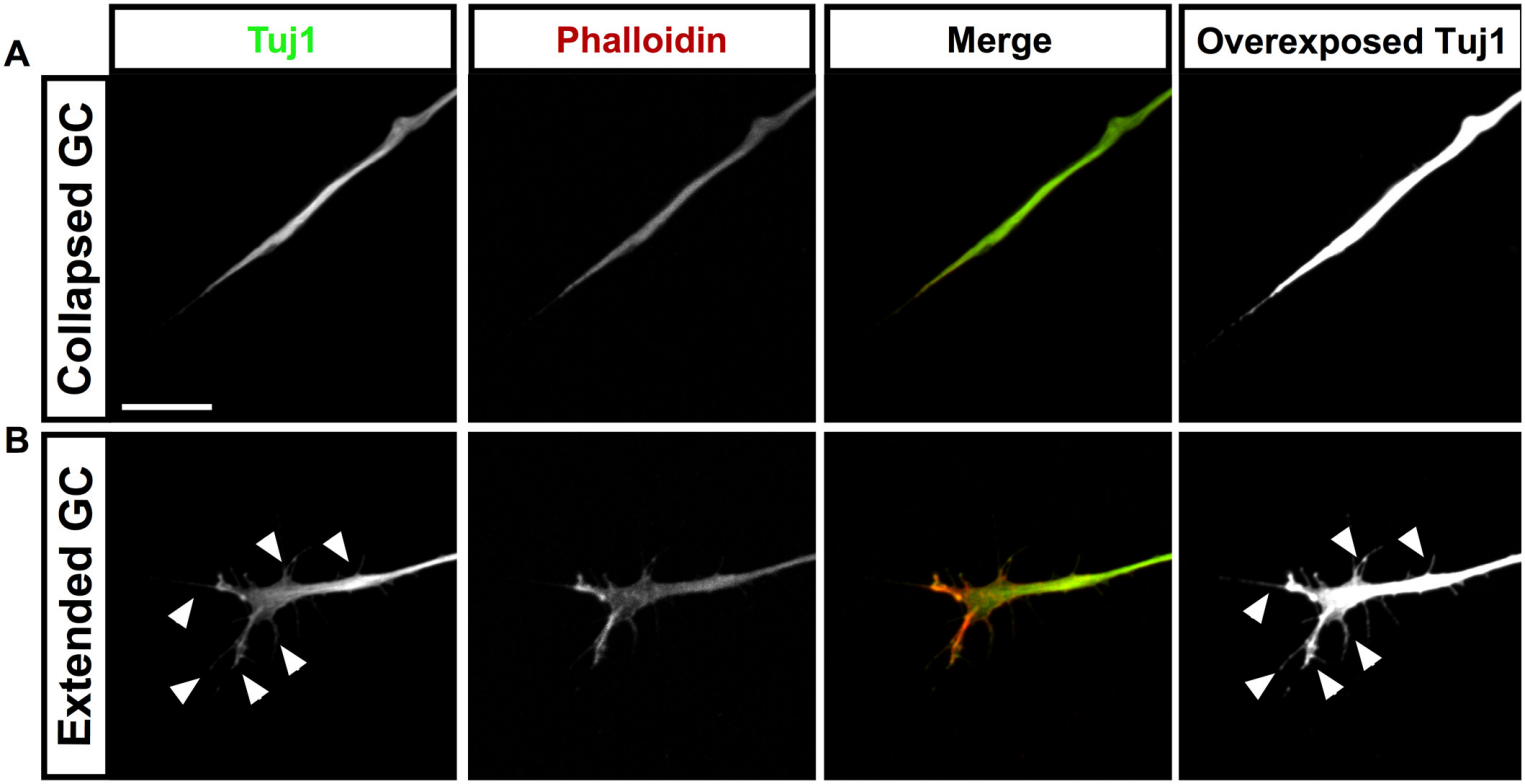
Tuj1 staining accurately reflects growth cone morphology. Co-staining of both collapsed (A) and extended (B) GCs with Phalloidin (red) and Tuj1 (green) shows that over-exposure of the Tuj1 channel accurately reflects the morphology of growth cones, down to the level of individual filopodia (arrowheads), with very a high signal-to-noise ratio. Scale bar in A represents 25μm.

Fertilized chick eggs (Couvoir Simetin, Mirabel, QC) were stored for a maximum of 1 week at 18°C, incubated at 38°C and staged according to standard protocols [25]. Explants of chick dorsal root ganglia (DRGs), or LMC neurons, were derived from Hamburger Hamilton Stage 26–27 embryos. Embryos were sacrificed by means of decapitation using forceps, which conforms to Canadian Council On Animal Care regulations. Spinal motor columns were identified as lateral bulges along open-book spinal cords, with DRGs lying dorsally beneath their folds; once removed from their embryos, the open book preparations were placed in Neurobasal media (Invitrogen). Sharp tungsten needles (World Precision Instruments) were used to cut these structures into smaller segments. 10–20 explants per treatment were then removed and cultured on laminin-coated plates (Nunclon, 20 μg/ml Laminin, Sigma) for 18 hours at 37°C in 5% CO2 in culture media [Neurobasal (Invitrogen), B-27 supplement (1:50, GIBCO), 0.5 mM L-Glutamate (Sigma-Aldrich), 25 mM L-Glutamine (GIBCO), and Penicillin-Streptomycin (1:100, Wisent)]. For dissociated cultures, 10–20 DRG were collected, and dissociated by enzymatic digestion using 25% Trypsin for 10 mins at 37°C, prior to enzymatic quenching with culture media, and dissociation by trituration through a glass-polished Pasteur pipette. Dissociated cultures were incubated for 48 hours. All cultures were fixed with 4% PFA (Fisher, in PBS) solution and permeabilized with 0.5% Triton X-100 (Fisher, in PBS). Immunostaining was performed by incubating plates with 1:1000 mouse Tuj1 (Covance) followed by a 1:1000 donkey anti-mouse secondary conjugated to AlexaFluor^®^ 488 (Life Technologies), both at room temperature for 1 hour each, with prior blocking by 1% bovine serum albumin (BSA; Sigma Aldrich) in PBS with 0.5% Triton X-100 (Sigma Aldrich). To verify that Tuj1 immunostaining accurately reflected GC morphology, we performed co-staining using Phalloidin (Fig 1, Life Technologies). For both collapsed (Fig 1A) and extended (Fig 1B) growth cones, over-exposed Tuj1 labelling (green) filled the growth cone to a greater extent as phalloidin (red) with little background, indicating that this treatment is suitable for detection and analysis of growth cone morphology. For DRG collapse experiments, explants were treated for 30 minutes with 1 μg/mL recombinant mouse Netrin-1 (R&D Systems) dissolved in motor neuron media, prior to fixing at 37°; controls were treated with 1 μg/mL Fc dissolved in motor neuron media. Samples were coverslipped and mounted with Mowiol (Millipore).

### Imaging and Analysis

Images were acquired as tiled 20X composites using a Zeiss Axio Observer Z1 microscope with a CSU-X1M dual cam 5000 spinning disk and the Zen 2012 image acquisition software. 5–10 explants were selected per plate at random and all plates were blinded. Statistics for the Finder Evaluation were performed using Microsoft Excel for Mac 2011. Conographer was primarily run on a PC running 64-bit Windows 8.1 Pro with Java 1.8 and ImageJ v1.49u as well as a Mac running 10.7 OS X Lion, Java 8 for Mac OS X (update 45) and ImageJ 1.49u. Statistics in Matlab (MathWorks) were performed on a Macbook Pro with 10.7 OS X Lion, with Matlab 2013.

### The Conographer algorithm

**Fig 2:**
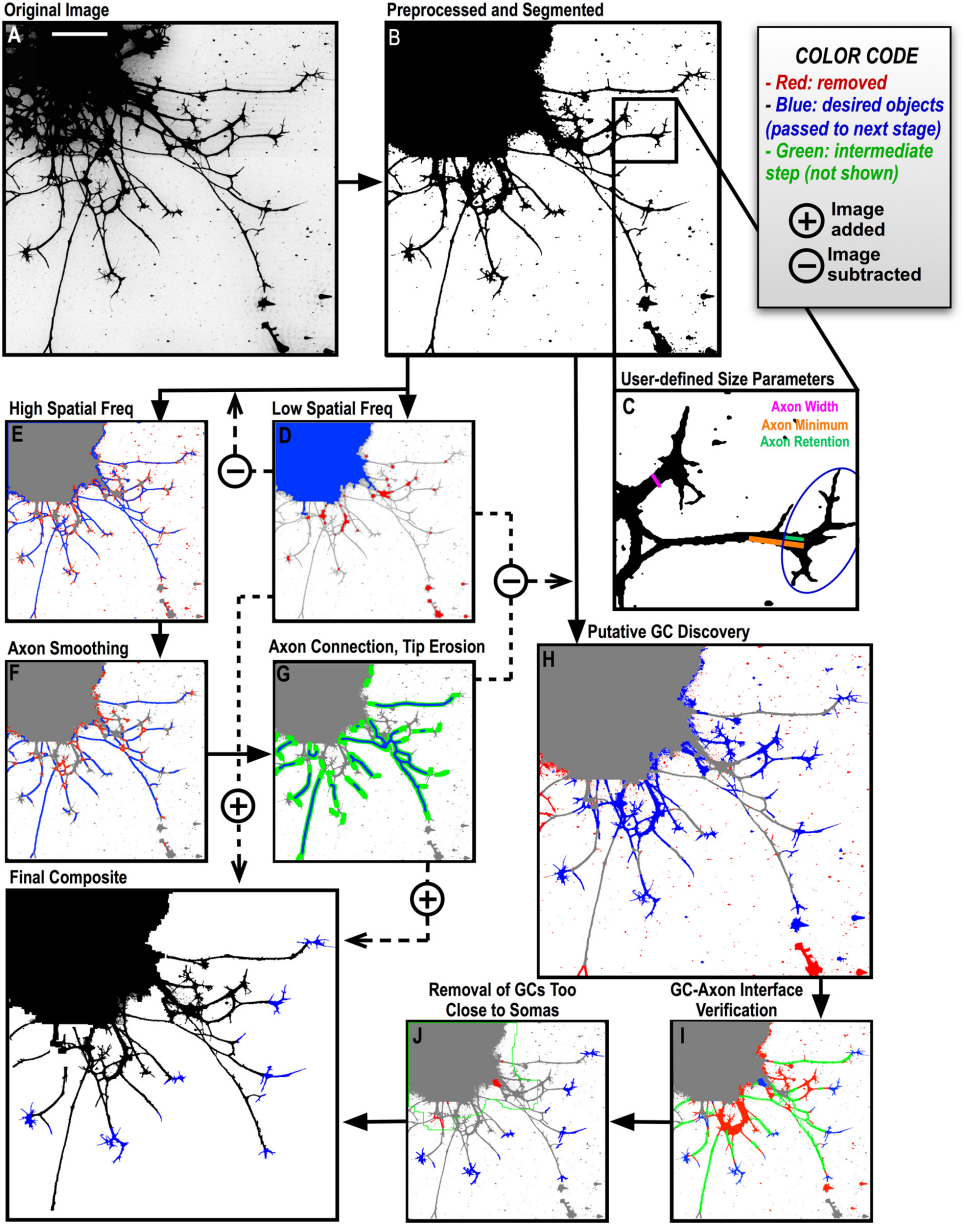
Conographer Description. A) A sample chick LMC explant with an overexposed anti-tubulin antibody stain. B) Basic preprocessing (Despeckling) and thresholding to segment the image into foreground objects (black) and background (white). C) Zoom of 2 GCs used to set the user’s three spatial parameters: Axon width (blue) corresponding to the expected upper bound width of an axon; Axon minimum (green) showing the minimum accepted length for an axon, such that shorter objects are assumed to be filipodia or other structures instead; and Axon Retention (red), the length of axon desired to be retained, measured retrogradely from the GC core branch point (or tip if there are no branches). D) The low spatial frequency image, created by eroding the segmented version by the Axon Width value to leave only objects of greater minimal diameters. In this case, the cell bodies of the explant are found as they are large enough to meet the size criteria (blue) while everything else is removed (red); in a dissociated culture, somas would be found with the presence of a nuclear stain such as DAPI instead. E-J) Conographer’s main steps: spatial frequency filtering to identify axons and cell bodies, axon processing to expose putative GCs, and two verification steps to eliminate false positive axons and GCs. Discarded structures are colored red, those which are passed to the next step blue, and green demarcates sub-steps not shown. E) The high spatial frequency image created by subtracting the low frequency image from the segmented original (blue), consisting primarily of axons, thin GC processes, and noise; most of the latter two are removed with a size filter (red). F) Remaining spindles are cleared from the axons, as are other branch points and small branches (below the Axon Minimum length), to assure that no GCs remain on the image (red), leaving only uninterrupted stretches of putative axon (blue). G) Axons are dilated to connect disparate sections (green), and then eroded to the blue objects. This tip erosion will expose GCs in the next step. H) The explant cell bodies and axons of steps (D) and (G) are subtracted from the segmented original to expose noise particles, previously-unfound axon segments, and GCs. Another simple size filter removes very small structures, as well as those contacting the image edge (red). I) First verification: Putative GCs are checked against the axons (green). Those with more than 1, 1 inappropriately large, or zero axon interfaces are assumed to be parts of axons or, in the latter, noise, and are discarded (red). Those with one appropriately sized interface have that interface expanded until either the GC core or tip is reached, or the previously specified Axon Retention distance is reached. J) Second verification: The area around the cell bodies is scanned to remove too-close GCs (red); this is important as growth-cone-like objects adjacent to the cell bodies will not have been removed based on axon interfaces. K) The macro output: GCs (blue), Axons (green), and Cell Bodies (red).

The Conographer algorithm is divided into 4 main sections: pre-processing and segmentation, spatial filtering, verification, and GC measuring. Conographer is designed to process RGB color, grayscale, or (if the user intends to segment manually) binary TIFF images. Upon start-up, after providing the paths for input images and output data, 3 randomly-selected images are used to make a montage with which the user sets pre-processing and thresholding values. Pre-processing options include Contrast Limited Adaptive Histogram Equalization (CLAHE) [26], which enhances and effectively normalizes contrast across the image, and "Despeckle", which applies a small blurring kernel to smooth over background noise. These are made available to improve segmentation in poorer-quality images, but should be left at zero unless the image has a particularly low signal-to-noise ratio; in these cases processing values should be incrementally increased until an adequate binary image can be obtained, in order to minimize distortions to the image. As the verification and measurements Conographer performs are carried out on the post-processed image, these parameters and the thresholding value must be chosen carefully (Fig 2B). After segmentation, the user provides 3 size related parameters, either by typing them in or drawing them on the example image montage (Fig 2C blue): Axon Width is the maximum diameter of an axon in the image; Axon Minimum is a cut-off length above which objects are counted as axons and below which they are assumed to be GC processes or other structures (Fig 2C green); and Axon Retained is the length of axon desired to be included in the GC measurements (Fig 2C red). The user can also specify a size threshold, above which objects are counted as explants and cell bodies, as well as the distance, from a cell body, that a GC must be in order to be identified as such. Finally, if cell bodies are to be identified by a nuclear stain such as DAPI instead of only size, which will generally be the preferred option for dissociated cultures where soma sizes can be near that of GCs, the user is also prompted with another randomly chosen montage of that stain’s channel, with which they can set its required threshold and size. A text file of these settings is created at the end of this step, which can be loaded in for later analysis to ensure consistency and preclude the need to repeat this initial step.

After the parameters are set, images are opened, pre-processed, and thresholded into binary forms. The second step of the algorithm splits the image into high and low spatial frequency binary masks via iterations of the “erosion” function (Fig 2D and 2E). In this section, all foreground objects are divided into putative cell bodies, axons, GCs, and noise particles. In the low spatial frequency image, putative explant cell bodies are identified based on their size or the presence of the specified nuclear stain; everything else is removed (Fig 2D blue). Axons are identified in the high-spatial frequency image as all objects smaller than the Axon Width diameter and larger than the Axon Minimum length, thereby discounting all small noise particles and filopodia (Fig 2E red). The resulting putative axon segments are then processed via binary Skeletonization, erosion, and dilation operations to remove branches and crossings (Fig 2F red), connect disparate parts (Fig 2G green), and erode their tips (Fig 2G blue). The built-in Fiji “Skeletonize” function creates a single-pixel wide topological skeleton of every binary object in the image, which consists of lines equidistant to the boundaries of the original shape. Finally, this axon mask is combined with that containing cell bodies/explants and subtracted from a binary copy of the original image, leaving only GCs and larger noise particles (Fig 2H blue). Objects with caliper diameters (defined as the greatest distance between two points on the object’s perimeter) below ½ of the Axon Minimum length are assumed to be noise particles or artifacts of the axon process steps and are discarded, as are objects touching the image edge (Fig 2H red). This section ends with the segmented image having been divided into 3 masks: putative GCs, Somas and Axons (blue structures in Fig 2D, 2G, and 2H).

Presumptive GCs prior to the verification steps correspond to misidentified segments of axons (such as varicosities or points in which multiple axons cross), large noise particles, or genuine GCs. The former two are filtered out based on the number and size of axon contacts; 2 or more contacts indicates a varicosity or axon crossing, and zero contacts indicates a noise particle (Fig 2I, red). Misidentified axon segments are added to the axon mask, and then each object there is checked to ensure that it contacts an explant or cell body. Acceptable GCs, which have only a single axon interface, have the ends of their connecting axons eroded until only the user-specified Axon Retention length remains, or until the GC tip or central domain is reached, defined by a broadening of the axon’s diameter above the user-specified Axon Width. As a final optional check, any GCs that fall within the specified distance from a cell body (Fig 2J green) are removed (Fig 2J red). Before the found GCs are measured, masks of the 3 structure classes are pseudocolored and saved as a stack, allowing users to visually asses the performance of the algorithm and easily perform additional measurements on cell bodies or axons if desired (Fig 2K). The binary GCs are then analyzed as described below. Scale bar in A represents 50μm.

### GC Finding and Collapse Evaluation

**Fig 3:**
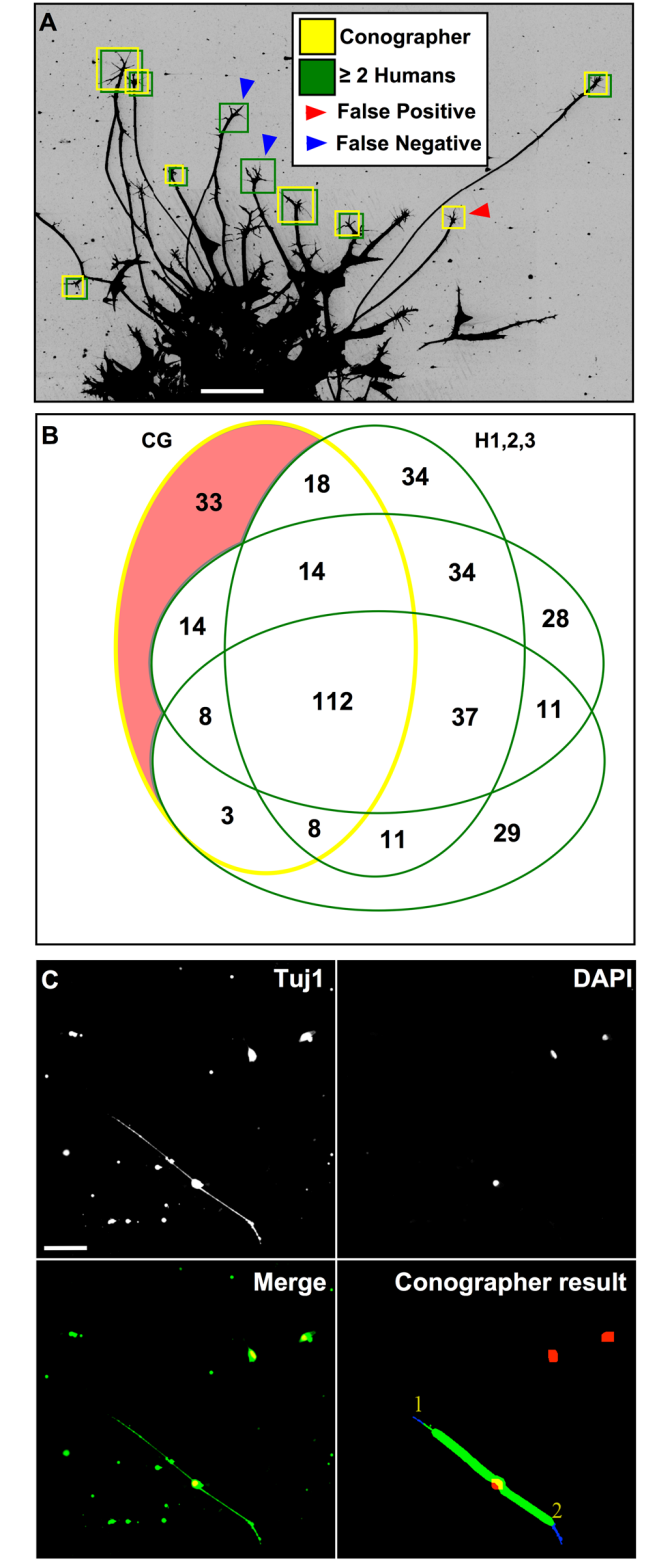
GC Finding Evaluation. A-B) Quantification of the Conographer’s (CG) GC-finding ability (yellow outlines) relative to the 3 human observers (H1, H2, and H3; green outlines) in a 7-image dataset. All observers found roughly the same number of GCs, (210, 268, 258, and 219 respectively). The blue portions of the diagram correspond to its 93 false negatives—ROIs identified by at least two humans and not Conographer–while the red portion corresponds to the 33 false positives found only by Conographer. By our criteria the images contained 235 true GCs, of which Conographer found 177 total, while the humans found an average of 218. C) In cultures of dissociated DRG neurons, Conographer identified growth cones (blue) associated with DAPI-positive nuclei (red) by Tuj1-positive axons (green). Scale bars in A and C represent 50μm and 40μm respectively.

Three human observers with prior experience analyzing collapse assays were given sets of 7 Fc-treated DRG explant images and asked to create regions of interest (ROIs) in Fiji around every GC that they would consider in a collapse assay. The observers were instructed to not include GCs if: 1) any of their processes touched other structures, 2) they stemmed directly from converging axons, or 3) they were less than ~100um from their explants. The four sets of ROIs (1 from Conographer, 3 from humans) were compared to each other with a custom Fiji macro, which calculated the proximities of their centroids; if they were within the Axon Minimum distance of each other, the ROIs were counted as overlapping. Cases in which two or more humans found a GC and Conographer did not were counted as false negatives, while cases in which Conographer found a GC that no humans did were counted as false positives (Fig 3A and 3B). Humans determined the collapse state of a GC by specific criteria; GCs with fewer than 3 filopodia and no obvious lamellipodia were classified as collapsed, while all other GCs were classified as extended. Additionally, we performed an experiment in which we dissociated DRG neurons, and incubated them for 48h to allow outgrowth of neurons (Fig 3C). Conographer discarded debris not associated with DAPI-stained nuclei and found growth cones with a similar size to the neuronal soma (Fig 3C).

### Conographer Measurements

**Fig 4:**
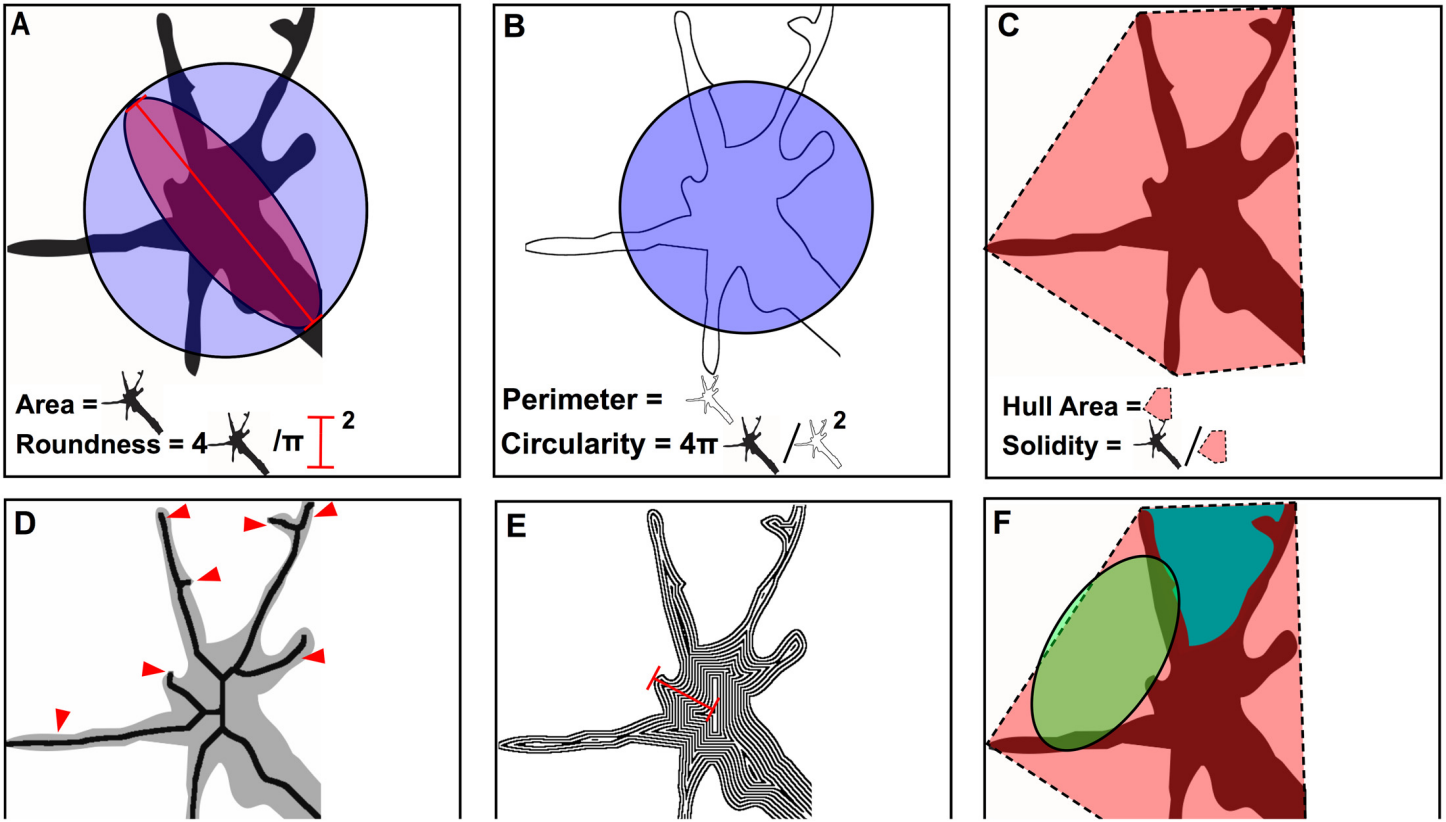
Ten measurements comprehensively describe GC morphology. A-C) Each panel contains a primary measurement and a derived ratiometric measurement with its graphical representation. A) The total GC Area and Roundness, which is derived by dividing the GC area (multiplied by 4) by a circle with the radius of the GC’s “major axis”. B) GC Perimeter, and Circularity, which is derived by dividing the GC area (multiplied by 4π) by the square product of the GC’s perimeter. C) Hull Area, which measures the area of a convex hull around the GC to approximate its spread, and Solidity, which measures the fraction of the convex hull covered by the GC. D-F) Non-ratiometric higher order measurements. D) Skeletonization, the total area of the skeletonized GC, as performed by the FIJI ‘Skeletonize’ function, and Branches, a count of the number of the skeleton’s terminal branches (arrowheads), excluding the axon. E) Thickness, which measures the number of binary erosions required to reduce the GC area to 0. F) Process Index, which approximates the number of processes as a count of domains between the GC’s perimeter and that of the convex hull, and Process Roundness, the average Roundness of these domains.

After isolating each verified GC (Fig 2K blue), Conographer performs the following measurements. The GC Area and Perimeter are measured as the GC’s total count of pixels and pixels on its edge, respectively (Fig 4A and 4B). Roundness and Circularity are calculated using the equations below (Fig 4A and 4B). Roundness essentially measures the inverse of the aspect ratio of the GC (Fig 4B); this measurement employs Fiji’s built in “Fit Ellipse” function to find the maximum diameter of the GC, from which the denominator of the ratio is derived. Circularity is measured as the GC area divided by that of a circle of the same perimeter as the GC perimeter, such that a perfectly circular GC will have a value of 1, with progressively lower values as the perimeter increases relative to area. This therefore is more of a measure of complexity; it would be possible for two GCs to have nearly identical Roundness values but different Circularity values if both had the same overall shape but one had more invaginations. We have included a description of the roundness parameter for completeness, as it is a measurement that Conographer makes, but this parameter is not considered in the further analyses made in this study, as it is less biologically relevant than circularity. Hull Area is defined as the area of a convex hull around the GC, a polygon that connects the most distal points of the structure, while Solidity is the ratio of its GC Area over the Hull Area (Fig 4C). Skeleton also approximates complexity by finding the total pixel count of the skeletonized GC, while Branches finds the number of branch points by counting pixels in the skeleton which are bordering more than two other pixels (Fig 4D). Thickness measures the “juiciness” of the GC by counting the number of erosions required to reduce its area to zero, such that a large GC with only thin filopodia would have a far lower value than an equally large one with thick lamellipodia. Finally, Process Index and Process Roundness measure the number of spaces between processes and their average roundness respectively, as a proxy for the number and shape of GC projections; these measurements and those relating to the convex hull have been shown to be effective for estimating process length [27].

$$Roundness=\frac{4\times area}{\pi \times Major\;axis^{2}}$$

$$Circularity=4\times \pi \times \frac{area}{perimeter^{2}}$$

### *K-means* clustering and calculation of concordance

Measurements derived from Conographer are exported into MATLAB (Mathworks) and processed as described. Z-scores are calculated using the formula below, which retains the variance and distribution of the data, while reducing all parameters to the same unit-less scale. Principal component analysis (PCA) is performed upon z-scores using the *pca* function in Matlab. Variables ranks are determined for each observer by either comparing means of GCs classified as ‘collapsed’ or ‘extended’, or by calculating the concordance between human and *k-means* clustering based upon single parameters. *K-means* clustering is performed using the *kmeans* function in Matlab. Labeled training sets were obtained via the manual ‘extended’ or ‘collapsed’ classification of 317 binary DRG-derived GC images found by Conographer from Fc-treated explants, by three observers. Concordance is determined as the percentage of GCs that are identified as being in the same state by both ‘observers’, be they human or *k-means* clustering. GCs at the edges of clusters are identified by finding the GCs with the highest Euclidian distance to the center of the cluster to which they do not belong. Because the *k-means* algorithm uses a random seed to determine cluster centers, each cluster does not always correspond to the same assignation (cluster 1 may be the collapse-associated cluster in one trial, cluster 2 in the second), however the associated assignation of each can easily be found by looking at the mean values of the cluster, as that with lower size and complexity-associated measurements corresponds to collapse.

$$Z=\frac{\chi -\bar{\chi }}{\sigma }$$

### Conographer Availability

A file containing Conographer can be downloaded from:

http://dx.doi.org/10.6084/m9.figshare.1565664

## Results

### Conographer accurately finds GCs

Firstly, to verify that over-exposed Tuj1 staining was suitable as a means of detecting the full complexity of a growth cone, we performed Tuj1 staining as described above, and co-stained with Phalloidin (Fig 1); overexposure of the Tuj1 channel accurately reflected the morphology of GCs, while maintaining a high signal-to-noise ratio. To verify the ability of Conographer to correctly identify GCs in images, we compared its performance to that of human observers. Three independent human observers agreed on a definition of a GC and identified GCs in a set of 7 images of explants of dorsal root ganglia (DRG) from HH St. 26–27 chick embryos that were cultured for 18-24h and fixed. Conographer was then used to identify GCs in the same images, and the results compared; these findings are shown in Fig 3. In terms of false positives, quantified as ROIs found by only a single observer, Conographer performed nearly indistinguishably from humans, with 84.3% accuracy relative to an average of 87.7% ± 0.7%. It found 75.3% of the “true” GCs (defined as being identified by at least two humans), while humans found 92.7% ± 0.6%.

### Comparison of collapsed and extended GCs

**Fig 5:**
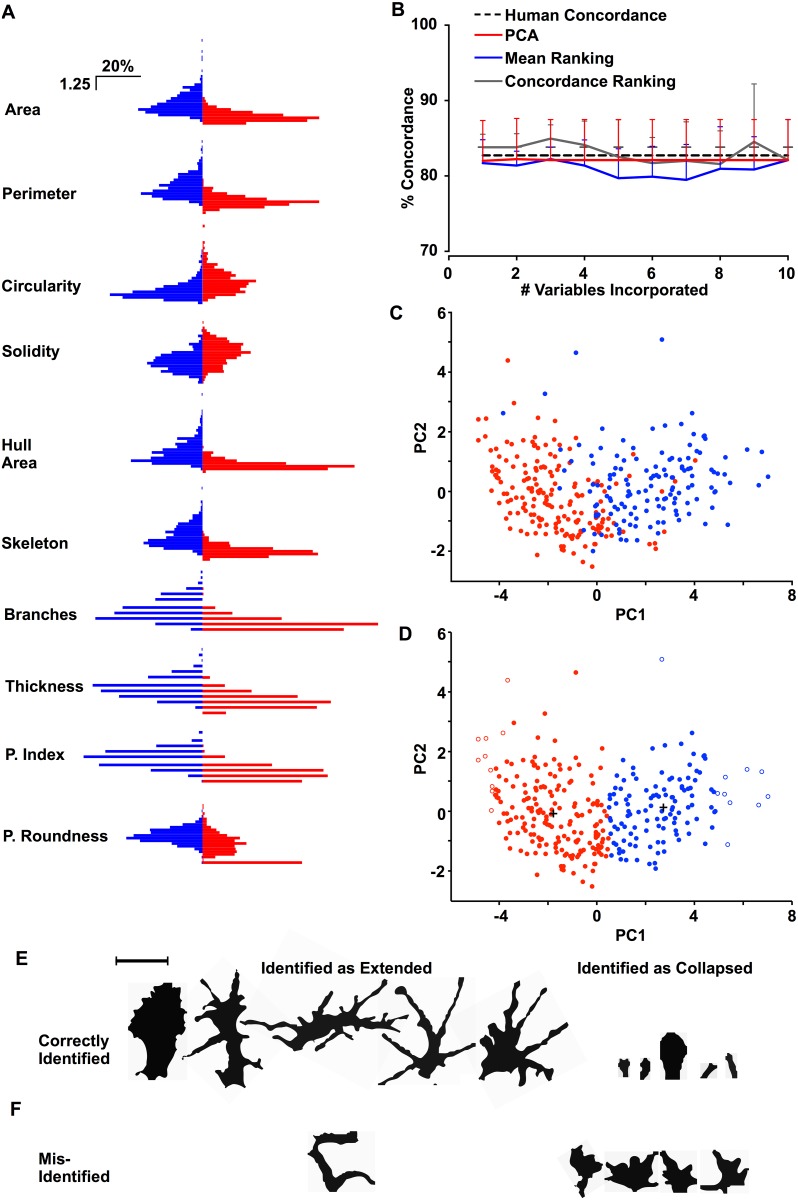
Comparison and assignation of growth cone collapse state. A) Distributions of Z-scores of the 10 Conographer-derived parameters in extended (blue) and collapsed (red) GCs. B) Concordance between human observers (black dotted line) and K-means clustering based upon PCA (red), and incorporation of multiple variables ranked by either their individual concordance (grey) or the difference between Z-score means (blue). C) Collapsed (red) and extended (blue) GCs, as determined by a human, plotted upon the first 2 principal components (PCs) derived from Z-scores. D) Collapsed (red) and extended (blue) GCs as determined by K-means clustering, plotted on the same axis as C; white-centred points indicate the GCs furthest from the centre of the cluster to which they do not belong. E) 5 extended and collapsed growth cones, as determined by K-means clustering. F) Growth cones misidentified by K-means clustering as either collapsed or extended.

Having verified Conographer as a useful tool to find GCs in images of cultured neurons, we verified the utility of each of the measured parameters of GC morphology. We examined 645 GCs that were classified as either extended or collapsed by a single human observer, and compared each parameter between extended and collapsed groups. Firstly we transformed each parameter to a Z-score, as described above; this retains the variance and distribution of each parameter and scales values to the same unit-less scale to allow for principal component analysis (PCA). We then compared the means and distributions of each parameter in collapsed or extended GCs (Fig 5A) using the Wilcoxon rank-sum (*ranksum* in Matlab) and Kolmogorov-Smirnov (KS; *ks2* in Matlab) tests for significance. As expected, all examined parameters were highly significantly different between collapsed and extended GCs by both tests (P<0.0001 in all cases, Table 1), reflecting the clear morphological differences between collapsed and extended GCs.

**Table 1:** Changes in parameters due to GC collapse.

	KS	RankSum
Area	5.82E-36	2.17E-40
Perim.	4.13E-34	9.51E-38
Circ.	1.12E-15	1.20E-19
Solidity	2.25E-11	4.81E-11
HullArea	1.50E-35	4.70E-38
Skeleton	2.37E-38	2.25E-40
Branches	3.11E-27	5.42E-32
Thickness	4.74E-24	4.90E-31
P.Index	9.09E-30	1.59E-35
P.Round.	6.41E-16	1.72E-15

KS = Kolmogorov-Smirnov

RankSum = Wilcoxon Rank-Sum (non-parametric) test

We then aimed to determine if these parameters could be used to assign a GC as being collapsed or extended in an automated, largely unbiased fashion. To determine which variables are most important in the assignment of GC collapse state, we examined a dataset of 317 GCs in which 3 independent observers had manually determined which GCs were collapsed and extended. Firstly, we compared the collapse state (i.e. collapsed or extended) of each GC between the three human observers, to determine how often humans agree with each other on the collapse state of given GCs. On average, the three observers were 82.75 ± 1.11% concordant with each other (Fig 5B, dotted line), and agreed unanimously on the identity of 83.91% of the GCs. To determine how effectively parameters derived from Conographer could ascertain GC collapse state in an unbiased fashion, we performed *k-means* clustering upon the 317 GCs for which collapse state had already been assigned; we repeated this process thrice, once for each observer’s collapse state assignment. After running the algorithm, clusters were associated with either collapse or extension based on the mean values of parameters for each; for example, if cluster 1 has lower area- and complexity-associated parameters but higher circularity, it to corresponds to collapse. Initially we performed clustering on only single variables and found a wide range of concordance between *k-means* and individual human observers, from 61.19% to 85.8%; ranking these variables by concordance revealed that no two human observers ranked parameters equally, suggesting that there is no hierarchy of Conographer parameters that definitively indicates collapse. We then performed clustering on multiple parameters; *k-means* clustering was performed 10 times for each observer, each time incorporating an extra parameter ranked by either the mean difference between collapsed and extended GCs (Fig 5B, blue), or the single-parameter clustering concordance described above (Fig 5B, grey). These analyses revealed that classifications using *k-means* clustering were as concordant with those of a human observer as a second human observer would be, suggesting that *k-means* clustering is a valid technique to determine GC collapse state.

Given that the ranking of parameters by concordance varied between human observers, precluding the use of a single set for the clustering, we employed PCA as a means to incorporate all of the parameters measured by Conographer into our analysis. Of the 17 parameters recorded by Conographer, we selected the 10 most biologically-relevant parameters recorded by Conographer for this and the following analyses: Area (GC size), Perimeter (a basic measurement of complexity), Circularity (a basic measurement of complexity), Solidity (how much a GC fills the area of its filopodial spread), Hull Area (the size of the environment which a GC can theoretically sample), Skeleton (a basic measurement of complexity), Branches (how many branches a GC makes), Thickness (how much thicker the GC is compared to its skeleton), Process Index (indicating the number of filopodia), and Process Roundness (a measure of the complexity of the GC periphery). The remaining parameters were generally rejected because they measured similar features as another, simpler, parameter; for example, Process Circularity was not used as it is a metric of the average complexity of each process domain, as it is too similar to the Circularity measurement. Performing PCA followed by *k-means* clustering with only the first 2 principal components resulted in classifications that matched 82.22 ± 5.41% with human observers, similar to the rates of concordance between humans. Given the variability in the weight given to individual parameters by different humans, and that PCA incorporates all of the Conographer parameters to some extent, we chose to use PCA for all further analyses.

We plotted the identified by one human as being extended (blue) or collapsed (red) GCs by the first two principal components (PC1, PC2, Fig 5C); it can be seen that collapsed GCs segregate away from extended GCs. We then re-plotted this data, with the collapse state replaced by cluster identity (Fig 5D); as can be seen in this graph, the plot is mostly similar to that derived from a human observer (Fig 5C). To verify the accuracy of GC collapse state assignment, we then examined the shapes of GCs belonging to each cluster by finding the GCs furthest from each cluster center (Fig 5D, empty circles), and identifying the ROI from which these measurements were derived; these GCs are shown in Fig 5E, and are clearly either extended or collapsed. We next looked at GCs that were misidentified by the clustering procedure, to find that humans unanimously agreed upon the collapse state for 83.91% of GCs, and that our clustering analysis disagreed with only 8.65% of these assignments (Fig 5F).

Together, these analyses demonstrate that Conographer-derived parameters differ significantly between collapsed and extended GCs. Additionally, analysis of these parameters based upon PCA and *k-means* clustering can assign collapse state identity to GCs with a similar degree of accuracy to humans.

### Analysis of Netrin-1-induced GC collapse

Having determined that Conographer-derived parameters are different between collapsed and extended GC, and useful in determining GC collapse state in a dataset with which collapse state has been previously determined for every GC, we examined if this were still the case upon the addition of a ligand with known collapsing activity. Netrin-1 is a secreted protein capable of attracting and collapsing different types of growth cones depending on their Netrin-1 receptor expression [28, 29]. Mutations in the gene encoding the repulsive Netrin-1 receptor Unc5c result in disrupted DRG axon projections in mice *in vivo* and Netrin-1 treatment results in the collapse of explanted DRG axons [30–32]. Thus, we expected to see an increase in the number of collapsed-cluster-associated DRG GCs detected by Conographer following Netrin-1 treatment. We cultured chick dorsal root ganglion (DRG) explants, as above, and treated them for 30 minutes with either Netrin-1, or Fc as a control. After treatment, we fixed, stained and imaged the neurons as in the previous experiments. GCs were found and measured using Conographer, then all measurements were pooled together for the purposes of calculating Z-scores.

**Fig 6:**
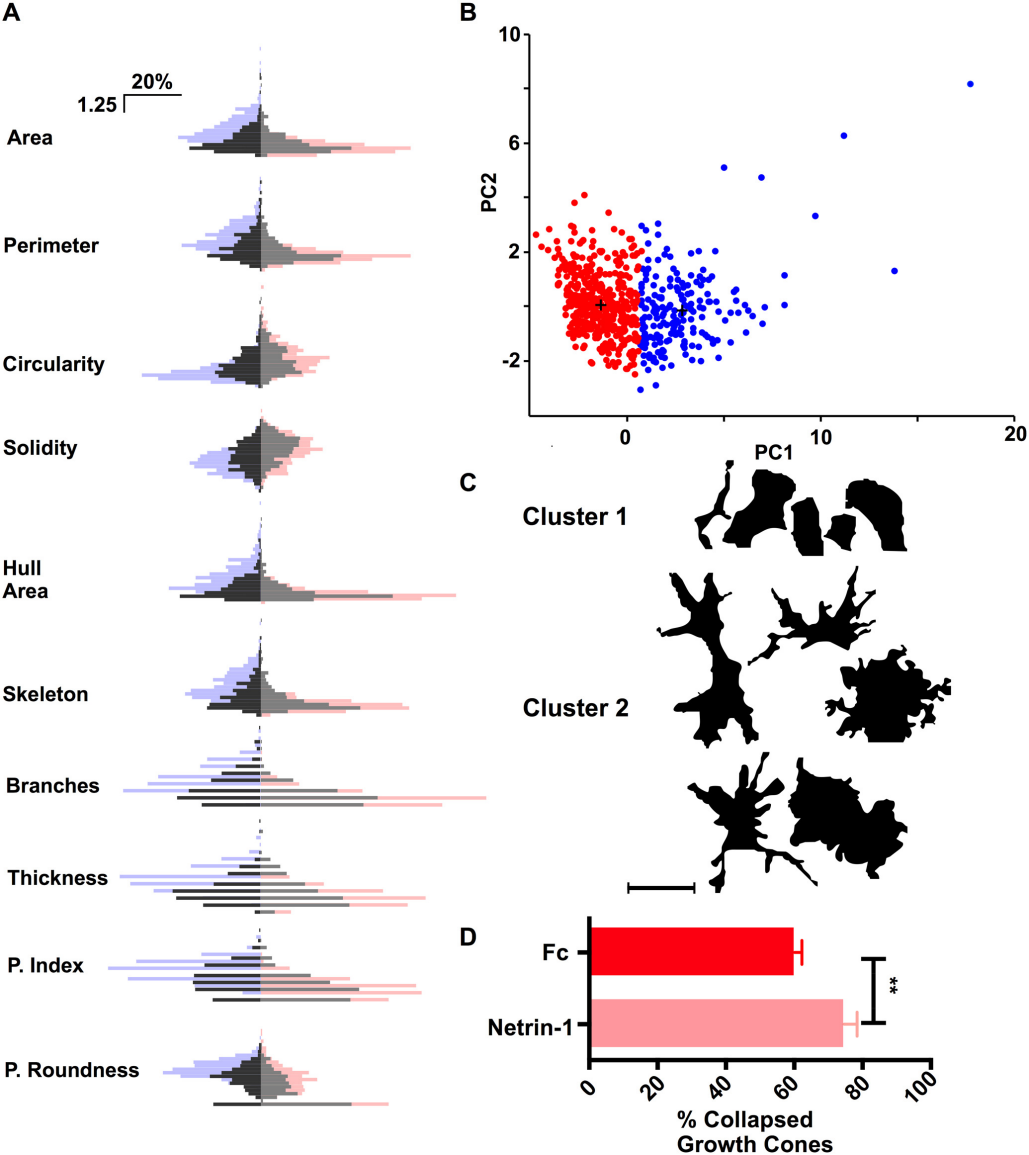
Conographer-derived parameters change during physiological collapse. A) Distributions of Z-scores of the 10 Conographer-derived parameters in Fc- (dark grey) and Netrin-1-treated (light grey) GCs superimposed over distributions of extended (pale blue) and collapsed (pale red) GCs. B) Plotting of GCs belonging to K-means derived clusters 1 (red) and 2 (blue) upon the first 2 principal components. C) Shapes of GCs belonging to clusters 1 (top) or 2 (bottom); cluster 1 GCs are simple, whereas growth cones belonging to cluster 2 are larger and more complex. D) Percentage of collapsed growth cones in explants from each treatment, as assessed by Conographer-derived measurements, PCA and k-means clustering. GC collapse percentage increased in the presence of Netrin-1 from 60.17±1.21% to 74.61±2.21% (p = 0.0046, Student’s two-tailed t-test). Scale bar in C represents 25μm.

Firstly, a dataset containing 621 growth cones derived from explants treated with either Fc or Netrin-1 was examined by 3 blinded human observers; 52.7 ± 7.91% of Fc-treated GCs were classified as collapsed, while 76.67 ± 3.72% of Netrin-1-treated GCs were classified as collapsed, indicating a strong collapse response induced by Netrin-1. The relatively high baseline collapse in this assay is likely due to the absence of NGF or NT3 growth factors in the motor neuron medium [33]. We then performed analysis of this dataset with Conographer; Fig 6A shows the distributions of each measured parameter in response to Fc (dark grey) or Netrin-1 (light grey). Performing Kolmogorov-Smirnov tests and Wilcoxon rank-sum tests revealed significant differences between treatments (Table 2) indicating that even in the absence of a 100% pure population of collapsed or extended GCs, Conographer-derived parameters are altered by collapse-inducing treatments. We next performed PCA followed by *k-means* clustering upon this dataset to automatically assign collapse identity to growth cones (Fig 6B). In Fc-treated explants, 58.3% of GCs were assigned to cluster 1; these GCs were smaller, and less complex than those in cluster 2 (Fig 6C), indicative of collapse. In Netrin-1-treated explants, the proportion of GCs in cluster 1 (i.e. collapsed) was 79.3%, reflecting the increase in collapse brought about by Netrin-1. Performing Conographer analysis upon a further 2842 GCs derived from two repetitions of the experiment yielded similar results; GC collapse percentage increased in the presence of Netrin-1 from 60.17±1.21% to 74.61±2.21% (p = 0.0046, Fig 6D). Together, these results indicate that Netrin-1 treatment induces changes in Conographer-measured GC parameters that are consistent with collapse, and that these are reflected by changes in GC collapse as determined by PCA and *k-means* clustering as well as human observers.

**Table 2:** Changes in parameters due to Netrin-1 treatment.

	KS	RankSum
Area	0.005341237	0.001266233
Perim.	1.64E-06	1.24E-06
Circ.	9.89E-05	8.35E-07
Solidity	9.73E-12	1.59E-15
HullArea	5.38E-07	2.72E-07
Skeleton	2.39E-08	1.70E-08
Branches	3.21E-08	9.47E-12
Thickness	0.034479029	0.014628886
P.Index	1.75E-07	9.55E-11
P.Round.	1.16E-12	9.24E-13

KS = Kolmogorov-Smirnov

RankSum = Wilcoxon Rank-Sum (non-parametric) test

## Discussion

### Conographer and k-means provide a more sophisticated collapse assay

The automated strategy of Conographer was developed to address key limitations of GC collapse assays, namely the laboriousness of larger analyses and subjectivity of defining and classifying GCs. Our algorithm provides a flexible and user-friendly tool for extracting GC structures from 2D images based on a series of shape-related criteria, thereby also providing a strict morphological definition. This standardization increases reproducibility and comparability of results from analyses with the same size parameter settings, and thus could facilitate the pooling of data between experiments, individuals, and even different laboratories. The inclusion of optional additional parameters adds some flexibility, such as restricting an analysis to neurons expressing a particular somatic marker in a dissociated culture, or investigating only GCs that have undergone a certain distance of outgrowth away from their somas. Basic pre-processing options are also provided, precluding the need for manual segmentation in images with relatively clear and even signal intensities. The masks of cell bodies, axons, and GCs output by Conographer allow visual assessments of its performance, and make each class of structure amenable to further analysis. If desired, there is also an option to save a “diagnostic mask”, a montage that displays all the major processing steps of the algorithm on a given image, to help users troubleshoot and optimize user parameters.

By reducing the dimensionality of the large output dataset with principal component analysis and subsequent *k-means* clustering, it is possible to accurately, objectively and automatically cluster GCs found by the Conographer into two or more clusters, and determine the percentage of GCs in each treatment group that are part of each cluster, as in a traditional collapse assay. Given that assaying concordance between humans and *k-means* clustering showed that each human observer ranked each parameter differently, we performed PCA, which incorporates a portion of each parameter into a new score, which can be used for clustering. Multivariate statistics-driven cluster analyses are employed frequently in genomics studies [34–36], and to a lesser extent have also been used for morphological classifications such as this [37, 38]. However, to our knowledge our PCA-driven approach is unique in that it requires no *a priori* assumptions or labeled “training set”; the clustering algorithm is capable of discriminating between collapsed and extended GCs from the principal components alone.

### Output masks and large datasets enable comprehensive analyses

Beyond its effectiveness in improving collapse assays, Conographer has the potential to facilitate a great number of other analyses in 2-dimensional explant and dissociated neural cultures. In particular, by partitioning images of cells into masks of their soma and axons it can be used to study the relationships of soma to their GCs. For example, to automate outgrowth assays in which the distance GCs travel from explants are quantified, one could find the GC centroids and the associated explant centroid, and then average the distance between them. If one were studying axon branching, one could find the area of axons for a given image and relate that to the number of associated GCs, to investigate how many GCs sprout per unit area of axon. Even more powerfully, because regions of interest corresponding to GCs are saved, one can relate the high-dimensional morphology measurements to molecules in additional channels. As an example, to test how ephrin-induced Eph clustering affects GC collapse, one could run Conographer and use the output ROIs on the channel with the Eph stain to analyze fluorescence intensity, then correlate this with changes in collapse-associated morphology parameters. The two features could even be combined, for example, by staining for a nuclear factor and correlating its intensity with GC branching. For use in microprinted gradient, or microfluidic gradient experiments, in which the angle of chemotropic cue gradient is known, one could examine how the morphology of a GC relates to not only the angle of the gradient, but also the position of the GC along a gradient [39]. Essentially, the modules of Conographer can facilitate nearly any study of the relationship between cell body, axon, or GC morphology, and fluorescence in each of these domains. By virtue of the entire algorithm being written in a clear modular form in the ImageJ macro language, even users with limited programming experience can make modifications such as these to automate otherwise laborious analyses.

Even without these modifications, the large datasets of GCs that Conographer is capable of producing are amenable to a number of other statistical analyses. Firstly, to identify different GC “modes” reflected in their morphologies, one could easily increase the number of clusters to see how the distribution of GCs shifts between them with different treatments, and qualitatively describe each cluster with the 10 measurement means. Indeed, recent experimental analyses have attempted subtler classifications, extending the dichotomy of collapse vs non-collapse by including intermediate categories [40], and Conographer would be well-suited for this kind of exploration. In this way, one could theoretically identify different functional cell types in a preparation of explants or dissociated cells in a completely unbiased way based on the responses of their associated GCs, thus providing evidence for population-specific responses. Beyond mathematical clustering, users can explore how individual parameters of interest (such as GC branching) change in response to various treatments, as well as more sophisticated factor analyses in which inferences can be made about underlying mechanisms driving changes in multiple parameters. Finally, because binary masks of the GCs themselves can be saved as well, one has the ability to build up arbitrarily large banks of GCs under a variety of conditions and treatments, permitting users to integrate results from multiple experiments and perform morphological analyses on datasets of unprecedented size.

### Conographer detects growth cone morphological changes induced by an axon guidance ligand in vitro

The correct targeting of sensory afferents to the spinal cord during development is dependent on Netrin-1:Unc5c signaling. In mice lacking either the ligand or its receptor, DRG axons aberrantly invade the spinal cord [30, 32]. The repulsive effects of Netrin-1 on chick and mouse DRG axons have been confirmed *in vitro* [31, 32]. In this study, treatment of chick DRG explants with Netrin-1 induced GC collapse as assessed by both human observers and Conographer. Whilst baseline collapse in our study may appear high, this is likely due to the absence of growth factors in the culture medium, as it has been previously reported that in the absence of NGF the rate of collapse in mouse DRG neurons is close to 80% [33]. Importantly, comparison to Lemons *et al*, [31], in which DRG GCs are treated with Netrin-1 to produce collapse, gives results that are qualitatively similar. These discrepancies are most likely to be a result of differing means of GC collapse assignation; in Lemons *et al*. it is the retraction or extension of the axon over the timecourse of Netrin-1 treatment that determines GC collapse state. Additionally, differences between growth substrata, growth factor concentration in the culture media, and the embryonic stage being examined will also contribute to differences in collapse rate. It is worth noting that the percentage of collapsed growth cones in control experiments can vary massively, with values from 10% [41] to 40% [42], to 80% [33], suggesting that more standardized, reproducible analyses, such as those provide by Conographer, would be useful.

Comparing our results with previous experiments serves to illustrate how Conographer facilitates higher-powered studies, as we were easily able to analyze samples containing thousands of GCs, where the more laborious timelapse methodologies are constrained to GC numbers in the double digits. Conographer’s suite of parameters are able to detect collapse in a more reproducible and quantitative manner than a subjective binary score by a human could ever do. While a properly blinded human scoring of a collapse assay allows experimenters to learn about a molecule’s repulsive properties, it fails to convey any information on GC characteristics both at baseline and after treatment, and is unable to say anything about a molecule’s effects on GCs at sub-collapsing concentrations, which often have powerful unexpected effects [39, 43].

Beyond the binary collapse assignations, the multivariate dataset can provide quantifiable information as to the morphological effects of experimental manipulations. In the case of Netrin-1 treatment, there are clear changes in the distributions of the 10 parameters used for the analysis which all seem to reflect its expected repulsive effect. In particular, striking changes can be observed for parameters associated with the length, number, and complexity of processes (Fig 6A, Circularity, Skeleton, Process Index, etc.), quantitatively demonstrating the retraction of filopodia and reduction in central domain mass that are associated with repulsion. This subtle characterization of the effects of Netrin-1 would be far more laborious for human observers to detect.

While a detailed analysis is outside the scope of this exposition, we envision Conographer being employed to make such subtle morphological comparisons between treatments. For example, in order to study the effect of a guidance cue on filopodia extension, one could compare only GCs of similar total area and number of processes while using measurements such as Skeleton, Hull Area, and Circularity as proxies for filopodia lengths. Conographer is also a perfectly suited tool for exploring, in a quantitatively rigorous way, a growth cone’s response to multiple overlapping cues, a paradigm that is undoubtedly the next page in the field of axon guidance research [44].

### Method caveats

As with any automated image analysis, one of the greatest sources of difficulty lies in the initial binary segmentation of the image into foreground and background. While sophisticated segmentation algorithms that employ contour detection and other strategies do exist, we have elected not to integrate them into Conographer. This was done in part to keep the algorithm relatively simple (and therefore amenable to adjustments by users), and fast, by applying global thresholds and basic contrast adjustments rather than individually segment each object, as would be the case with contour detection. Thus, most Conographer errors can be attributed to errors in segmentation, such as falsely attributing the disconnected end of an over-thresholded axon as a collapsed GC or failing to include a thin process separated by the thresholding in measurements. For this reason, it is of the utmost importance that care is taken to maximize the signal-to-noise ratio and minimize fluctuations in signal intensity in the stained structures; both of these concerns are addressed by use of the Tuj1 antibody, which gives excellent signal to noise ratio. Background noise may slow the algorithm down as particles are initially misidentified as GCs, but will not interfere with the validity of the actual measurements as the vast majority will be rejected in the verification steps. If global thresholding is distorting structures in a set of images too much, or if signal intensities vary significantly between images, it is advisable to manually threshold such images or employ more sophisticated segmentation algorithms before beginning analysis with Conographer.

Despite the possibility of segmentation errors, by overexposing the TUJ1 channel we were able to obtain and clearly segment fine processes associated with GCs and recapitulate the results of humans (Fig 1). Though GCs are generally thought to be identified by their high concentrations of F-actin, we were unable to use actin-staining phalloidin as a morphology reporter because Conographer requires strong signals in axons, where the intensity is much less. We elected not to build the algorithm around phalloidin in conjunction with another stain that marks axons, as that would have necessitated compensating for unavoidable differences in ratios of intensities between the two channels, and likely would create more variability in what are identified as GCs. Additionally, by requiring only a single channel, a wide variety of genetic and chemical tools can be used on living or fixed tissue, with all other channels free for additional stains. This being said, if a user is unable to find a way to clearly demarcate both axons and GC processes with a single channel, multiple channels can be combined prior to running Conographer.

### Speed and accessibility

Whilst use of Conographer does require the experimenter to capture images, this can be largely automated by use of microscopes with a motorized XY stage, with which the finding and measurement of GCs can occur within ~15s per explant image; even this time-per-explant could be reduced if Conographer is used in conjunction with high-throughput automated microscopy systems. Even if users decide to forgo the *k-means* based analysis, we have included an ImageJ macro that allows rapid collapse state assignment by experimenters, by simply presenting images of individual GCs and asking the user to classify them. Alternatively, users can also use the measuring module of the Conographer on pre-identified binary images of any structure. Conographer the measuring module, the GC finding module, and the macro to facilitate manual sorting are all freely available at http://dx.doi.org/10.6084/m9.figshare.1565664 or by communicating with the authors.

## Conclusion

In this study, we demonstrate the utility of the Conographer macro, which we have developed to automatically detect and measure GCs *in vitro*. We have shown that Conographer finds GCs with only a slight reduction in frequency, and with an insignificant number of false positives relative to humans. In addition, we demonstrate that the 10 parameters employed during our cluster analyses are distributed differently between collapsed and extended GCs, and that these parameters can be used to classify GCs as extended or collapsed using a *k-means* clustering method, with the same level of accuracy as human observers. Finally, we illustrate how the parameters measured by the Conographer are changed by application of physiological collapse-inducing ligands, resulting in measurable differences collapse state. Together, these results demonstrate the potential of the Conographer as a powerful tool for the analysis of GCs *in vitro*.
